# Predictive model of psychological factors influencing university students’ intentions and behavior regarding physical exercise—based on machine learning algorithms

**DOI:** 10.3389/fpsyg.2026.1737828

**Published:** 2026-04-13

**Authors:** Lihong Zhu, Fanjing Kong, Tianzhuo Liu

**Affiliations:** 1 School of Physical Education, Northeast Normal University, Changchun, China; 2 Northeast Normal University Library, Changchun, China; 3 Shenyang Normal University, College of Sports Science, Shenyang, China

**Keywords:** influencing factors, machine learning, physical activity, predictive models, university students

## Abstract

Insufficient physical exercise poses a substantial threat to the physical and psychological wellbeing of university students. However, participation in physical activity is shaped by a complex interplay of psychological and physiological factors. This study employed machine learning techniques to predict levels of physical exercise participation and to identify key influencing factors among university students. Questionnaire data were collected from students across multiple provinces in China. Five classification models—Multi-Layer Perceptron (MLP), Extreme Gradient Boosting (XGBoost), Random Forest, Gradient Boosting Decision Tree (GBDT), and Decision Tree—were constructed and evaluated using standard classification metrics on an independent test set. Among the five models, the GBDT exhibited the highest predictive accuracy. Feature importance analysis indicated that body weight and anxiety were the most influential predictors of physical exercise participation. Partial dependence plots revealed non-linear relationships between key psychological variables and exercise behavior, particularly in differentiating higher levels of physical activity. In addition, the decision tree model identified resistance to temptation as the primary decision node, followed by healthy habits and impulse control. By integrating the Multi-Process Action Control (M-PAC) framework with stress process theory, this study elucidates how psychological stress, self-regulatory capacity, and behavioral habits jointly influence physical exercise participation. The findings provide practical implications for the development of targeted interventions aimed at promoting sustained physical activity among university students.

## 1 Introduction

Physical exercise is widely recognized as an effective means of health promotion and plays a critical role in enhancing subjective wellbeing, fostering positive psychological attributes, and strengthening psychological resilience when performed at appropriate intensity, frequency, and duration. However, insufficient physical activity worldwide poses a considerable threat to both physical and mental health, even contributing to reduced life expectancy. The World Health Organization has classified insufficient physical activity as the fourth leading global risk factor for mortality (Santos et al., 2023). In China, the healthcare burden attributable to insufficient physical activity has increased steadily in recent years. According to the Global Status Report on Physical Activity (2022), approximately 1.4 billion adults worldwide do not meet the World Health Organization’s recommended physical activity guidelines, defined as at least 150 min of moderate-intensity or 75 min of vigorous-intensity physical activity per week (World Health Organization, 2010). This demographic accounts for approximately 27.5% of the global population (World Health Organization, 2022b). Accordingly, promoting participation in physical exercise to improve physical and mental wellbeing has become a key public health priority within the strategic framework of the Healthy China initiative (World Health Organization, 2022a). Physical exercise behavior is shaped by multiple interacting factors, with increasing research attention devoted to the intention–behavior relationship (Jingyi and Sheng, 2024). Although most individuals report a clear intention to engage in physical exercise, a substantial proportion—estimated at approximately 47.6%—fail to translate this intention into actual behavior (Feil et al., 2023). This discrepancy is commonly referred to as the intention–behavior gap.

Exercise behavior is a multi-stage and dynamic process, encompassing the full trajectory from preparation and initiation to long-term maintenance (Gardner et al., 2016). Accordingly, stage-specific intervention strategies are required to effectively promote physical activity across different phases of behavior change. Previous research indicates that emotional processes play a more prominent role during the initiation phase of exercise, whereas rational processes are more influential in the maintenance phase. Interventions grounded in dual-process theory may therefore be particularly effective in enhancing the persistence of exercise behavior (Meiting and Liancheng, 2024). Previous studies have explored the relationship between exercise intention and behavior, with a primary focus on predictor selection, model development, and the interplay between internal psychological and external contextual factors (Jingyi and Sheng, 2024; Liancheng et al., 2025). First, much of the existing literature relies on linear statistical approaches or a limited set of predictors, which may be insufficient to capture the complex and potentially non-linear relationships among multiple psychological determinants of exercise behavior. Second, although the multi-stage nature of physical exercise participation has been widely acknowledged, few empirical studies have examined whether the relative importance of psychological predictors varies across different levels of exercise participation. Third, prior research has often been guided by a single theoretical framework, which may constrain a more integrated understanding of how self-regulatory and stress-related psychological processes jointly shape exercise behavior.

To address the aforementioned issues, an integrated perspective is required to examine the complex interaction pathways among self-control, emotion regulation, anxiety and depressive states, and sociodemographic variables (e.g., gender and household registration status). This approach allows for a more comprehensive elucidation of the mechanisms underlying the translation of exercise intention into actual behavior among university students. On this basis, the present study collected cross-sectional data from university students across multiple provinces in China in May 2023. Using five machine learning models—multilayer perceptrons (MLP), extreme gradient boosting (XGBoost), random forests, gradient-boosted decision trees (GBDT), and decision trees—this study systematically examined psychological and sociodemographic factors associated with physical activity. The aim was to identify key predictive variables and explore their underlying associative pathways through data-driven approaches, thereby providing theoretical and empirical support for promoting physical exercise behavior among university students.

### 1.1 Relevant research and theoretical foundations

The positive association between physical exercise and mental health is well established. However, individuals with poorer mental health often experience greater difficulty maintaining regular physical activity due to emotional distress, cognitive burden, and reduced motivation. Although established behavior change theories, such as the Theory of Planned Behavior and the Health Belief Model, emphasize intention formation as a prerequisite for action, substantial empirical evidence indicates that intentions alone are frequently insufficient to sustain behavioral engagement, giving rise to the widely observed intention–behavior gap. To examine patterns of intention–behavior translation, this study adopts an integrated analytical framework drawing on the Multi-Process Action Control (M-PAC) model and Stress Process Theory. The M-PAC model offers a process-oriented perspective on exercise engagement, while Stress Process Theory highlights how stress-related psychological states, such as anxiety and depression, are associated with variations in self-regulation and physical activity participation. Together, these perspectives provide a coherent framework for organizing psychological and situational correlates of exercise intentions and behaviors among university students.

Within this analytical framework, the term “Temptation to Not Exercise” is used as a descriptive label to organize psychological factors associated with resistance to physical activity participation under real-world conditions. Consistent with previous research, it refers to internal and external influences encountered during exercise that are associated with behavioral interruption or disengagement (Hausenblas et al., 2001). In the present study, this term is not treated as an independent construct, mediator, or composite variable. For analytical clarity, variables grouped under this label are organized into two distinguishable aspects: affective temptation and competitive temptation. Affective temptation reflects negative emotional experiences related to physical activity participation, including anxiety, depressive mood, and individual differences in emotion regulation strategies. These variables describe the extent to which emotional discomfort or maladaptive emotional responses are present during or in anticipation of exercise. From the perspective of established behavioral theories, such emotion-related factors are consistent with prior findings in the Theory of Planned Behavior, which highlights the role of attitudinal evaluations and perceived behavioral constraints in exercise-related decision-making (Ajzen, 2001). However, rather than modeling these components explicitly within a TPB framework, the present study treats them as empirically observable correlates of exercise participation and organizes them descriptively within the analytical framework.

Competitive temptation refers to alternative demands that compete with exercise for limited time, motivation, and self-regulatory resources, such as academic workload or leisure activities. Within this aspect, self-control reflects the individual’s capacity to prioritize goal-directed behavior when faced with competing alternatives. Existing evidence indicates that higher self-control is associated with greater behavioral stability and more consistent engagement in health-related behaviors, including physical activity. The psychological variables representing affective and competitive temptations—specifically anxiety, depression, emotion regulation strategies, and self-control—constitute the core feature set of the present study. These variables are measured using validated psychometric instruments and are entered individually into the machine-learning models as separate predictive features, providing a clear and empirically grounded basis for subsequent analyses of physical exercise behavior.

### 1.2 Physical exercise behavior and the multi-process action control model (M-PAC)

In health behavior research, physical exercise has commonly been examined through intention-based theoretical perspectives, which view behavioral intention as the most proximal predictor of physical activity (Kaushal et al., 2017; Rhodes and Yao, 2015; Vallerand et al., 2016; Vallerand et al., 2017). Representative frameworks include the Theory of Planned Behavior (TPB), the Theory of Reasoned Action (TRA) (Sheppard et al., 1988), and Social Cognitive Theory (SCT) (Young et al., 2015). Across these approaches, cognitive evaluations, attitudes, and perceived social influences are emphasized as central to the formation of exercise intentions.

However, empirical evidence consistently shows that intention alone does not guarantee behavioral enactment. Despite expressing clear intentions to engage in physical activity, a substantial proportion of individuals fail to translate these intentions into actual exercise behavior. Approximately 47.6% fail to successfully translate these intentions into actual behavior (Feil et al., 2023), a phenomenon widely described as the “intention–behavior gap.”

To address this limitation, the Multi-Process Action Control (M-PAC) model extends traditional intention-focused perspectives by incorporating post-intentional processes relevant to behavioral execution and maintenance (Rhodes, 2017). Specifically, M-PAC distinguishes three complementary processes: a reflective process related to the formation of intention, a regulatory process involving deliberate self-regulation strategies during early behavior enactment, and a reflexive process characterized by increasingly automatic patterns that support long-term behavioral maintenance (Tang et al., 2024). By shifting attention from intention formation alone to the broader implementation and maintenance of physical activity, M-PAC provides a coherent theoretical framework for identifying key psychological correlates of exercise behavior and informing subsequent empirical modeling.

### 1.3 Physical activity stress process theory

Although the benefits of physical exercise for both physical and mental health have been well established, substantial barriers to participation persist in practice, particularly among populations at elevated risk of mental health problems. Empirical evidence indicates that individuals with mental disorders such as depression and anxiety generally report lower levels of physical activity (Vancampfort et al., 2017), while sedentary behavior is more prevalent in these groups (Schuch et al., 2017).

Characteristic symptoms—including low mood, fatigue, reduced motivation, and heightened perceived stress—substantially increase the difficulty of initiating and maintaining engagement in physical exercise (Pomp et al., 2010; Glowacki et al., 2017). In addition, factors such as physical comorbidities (e.g., obesity), low self-efficacy, financial constraints, and limited social support further restrict participation in physical activity among individuals with mental health difficulties (Firth et al., 2016; World Health Organization, 2022b).

Stress Process Theory provides an important conceptual perspective for understanding these barriers (Xiangrong, 2023). This framework suggests that exposure to stressors may elicit a range of psychological responses, often manifesting as emotional states such as anxiety and depression (Junlin, 2017). Anxiety is typically characterized by tension and apprehension toward perceived threats, whereas depression involves persistent low mood, diminished self-confidence, and loss of pleasure. These emotional states may reduce motivation for physical activity and compromise self-regulatory capacity, thereby impeding both the initiation and continuation of exercise behavior.

In the present study, Stress Process Theory is integrated with the M-PAC framework to complement its explanation of the intention–behavior gap from emotional and cognitive perspectives. This integration highlights the role of anxiety and depression as key psychological factors that may disrupt behavioral regulation and the development of stable exercise patterns. Together, these perspectives support the construction of a more comprehensive research framework and provide theoretical justification for identifying psychological correlates of exercise behavior, particularly in relation to sustained physical activity and mental wellbeing among university students.

### 1.4 Research on physical activity participation based on machine learning

Currently, research on physical activity behavior faces challenges related to the multidimensional nature of influencing variables, complex interrelationships among psychological factors, and the limitations of traditional analytical approaches. Although theory-based behavioral frameworks, such as the M-PAC model and stress process theory, provide structured perspectives for understanding exercise-related psychological processes, more efficient data-driven methods are required to translate theoretical insights into robust behavioral prediction. Machine learning approaches, owing to their strong nonlinear modeling capacity and ability to handle high-dimensional features, are particularly suitable for constructing stable and interpretable predictive models based on multidimensional and interacting psychological and behavioral variables (Hastie et al., 2009). These methods offer clear advantages over traditional statistical techniques in addressing population heterogeneity and reducing redundancy among correlated predictors.

In the domain of physical activity prediction, previous studies have applied machine learning at different analytical levels. For example, Lee et al. (2023), integrated 28 categories of social, economic, and physical environmental variables to construct predictive models of regional physical activity patterns. Similarly, Liu et al. (2023) combined university students’ interest in physical education with autonomy-related variables and compared predictive performance across seven machine learning algorithms. In addition, research addressing the intention–behavior gap has explored computational models incorporating factors such as self-efficacy, social norms, and digital device use. Within the intersection of mental health and physical activity, machine learning techniques have further been used to identify associations between activity patterns and depressive symptoms (Nawrin et al., 2024).

However, existing predictive models predominantly emphasize external environmental factors or isolated psychological indicators and rarely incorporate theory-informed psychological variables in an integrated manner. In particular, variables highlighted in the M-PAC framework and stress process theory—such as self-control, emotion regulation strategies, anxiety, and depression—have seldom been jointly examined within a unified predictive framework. This limits both the theoretical interpretability and the applicability of existing models to specific populations.

On this basis, the present study adopts M-PAC and stress process theory as guiding frameworks for variable selection. Key psychological variables—including self-control sub-dimensions, cognitive reappraisal and expressive suppression strategies, and anxiety–depression levels—are systematically incorporated to construct a categorical prediction model of physical activity levels among university students. Five machine learning algorithms—multi-layer perceptron (MLP), XGBoost, random forests, gradient-boosted decision trees (GBDT), and decision trees—are employed for model training and comparison. The study aims to: (1) evaluate the predictive contribution of theory-informed psychological variables; (2) identify key psychological correlates associated with physical activity participation; and (3) establish an optimized classification model to support targeted health promotion among university students.

## 2 Research methods and data

### 2.1 Data sources

This study employed a convenience sampling strategy to recruit university students from multiple provinces in China through an online questionnaire survey. The sampled locations included Changde City (Hunan Province), Jingdezhen City (Jiangxi Province), Wenzhou City (Zhejiang Province), Langfang City (Hebei Province), Yinchuan City (Ningxia Hui Autonomous Region), Heyuan City and Guangzhou City (Guangdong Province), and Guiyang City (Guizhou Province). Together, these sites span the Northern, Central, Eastern, Southern, and Northwestern regions of China, allowing the inclusion of participants from varied geographical contexts within the constraints of convenience sampling. The questionnaire consisted of sections assessing demographic characteristics, self-control, emotion regulation strategies, and symptoms of anxiety and depression. Data quality screening was conducted using multiple criteria, including questionnaire completion time, consecutive identical responses, extreme outliers, and pre–post consistency checks.

A total of 1,550 questionnaires were distributed via an online research platform, of which 1,253 were returned (response rate: 84%). After quality screening, 1,018 questionnaires were retained as valid. The final sample included 514 male (50.4%) and 504 female (49.6%) participants; 508 undergraduate students and 510 vocational college students; and 338 urban and 680 rural respondents, indicating a relatively balanced gender distribution and diversity in educational and residential backgrounds.

It should be noted that the use of convenience sampling limits the generalizability of the findings. The sample is not statistically representative of the national university student population in terms of provincial proportions or institutional types, as regional sample sizes were influenced by researcher access and campus cooperation. However, recruiting participants from multiple provinces reduces the likelihood that the observed results reflect a single local context. Accordingly, the subsequent machine learning analyses focus on identifying psychological and behavioral patterns within a geographically diverse, though not nationally representative, sample. Future studies employing stratified or probability-based sampling designs are warranted to further examine the robustness of these findings.

### 2.2 Data collection

#### 2.2.1 Personal basic information

The research project collected respondents’ demographic and background information, including gender, age, educational attainment, institution level, field of study, household registration status, and body weight, to characterize the basic profile of the sample.

#### 2.2.2 Predictor variables for physical activity

##### 2.2.2.1 Physical activity level scale

Physical activity was assessed using the Chinese version of the Physical Activity Rating Scale developed by Liang (1994), which has been widely used among Chinese college student populations. The scale evaluates physical activity over the past month across three dimensions: exercise intensity, duration, and frequency. Each dimension is rated on a 5-point Likert scale, and the overall physical activity score is calculated as the product of intensity, duration, and frequency, with higher scores indicating greater exercise volume.

Based on established cut-off criteria for this instrument, physical activity levels were classified as low (20), moderate (20–42), or high (≥43). In the present study, physical activity level was operationalized as a three-category ordinal outcome variable for classification modeling, a procedure commonly adopted in prior behavioral prediction studies to enhance interpretability. The scale demonstrated acceptable internal consistency in the current sample (Cronbach’s *α* = 0.730).

Despite its early development, this scale continues to be applied in contemporary large-scale surveys of Chinese college students due to its simplicity and stable scoring framework.

##### 2.2.2.2 Depression rating scale

Depression was assessed using the Self-Rating Depression Scale (SDS) developed by Zung et al. (1965). The scale consists of 20 items rated on a 4-point Likert scale ranging from “never or very rarely” to “almost all or all of the time,” with higher scores indicating higher levels of depressive symptoms. In the present study, the scale demonstrated acceptable internal consistency (Cronbach’s *α* = 0.771).

##### 2.2.2.3 Anxiety rating scale

Anxiety was assessed using the Zung Self-Rating Anxiety Scale (SAS) developed by William and Zung (1971). The scale comprises 20 items rated on a 4-point Likert scale ranging from “never or rarely” to “most or all of the time,” with higher scores indicating greater levels of anxiety. In the present study, the scale demonstrated good internal consistency (Cronbach’s *α* = 0.850).

##### 2.2.2.4 Self-control assessment scale

Self-control was assessed using the Revised College Student Self-Control Scale developed by Shuhua and Yongyu (2008). The scale comprises 19 items across five dimensions—impulse control, healthy habits, resistance to temptation, focus on study (or work), and moderation in leisure activities—rated on a 5-point Likert scale, with higher scores indicating greater self-control. In the present study, the scale demonstrated acceptable internal consistency (Cronbach’s *α* = 0.803).

##### 2.2.2.5 Emotional regulation strategies questionnaire

Emotion regulation strategies were assessed using the Emotion Regulation Questionnaire (ERQ) developed by Gross and Thomson (2007). The questionnaire comprises 10 items across two subscales—cognitive reappraisal and expressive suppression—rated on a 7-point Likert scale ranging from 1 (strongly disagree) to 7 (strongly agree), with higher scores indicating a greater tendency to employ the corresponding emotion regulation strategy. In the present study, the scale demonstrated good internal consistency (Cronbach’s α = 0.863).

#### 2.2.3 Distinguishing between validity and confirmatory factor analysis

Confirmatory factor analysis (CFA) was conducted using AMOS 29.0 to examine the discriminant validity among five latent constructs: Physical Activity Level, Depression, Anxiety, Self-Control, and Emotion Regulation Strategies.

The five-factor model demonstrated an acceptable fit to the data (*χ*^2^/df = 2.923, RMSEA = 0.061, CFI = 0.915, TLI = 0.920). Moreover, the five-factor model showed a better fit than alternative models in which latent constructs were combined. These results indicate that the five constructs are empirically distinct and exhibit good discriminant validity.

### 2.3 Machine learning model construction process

#### 2.3.1 Data pre-processing

Data preprocessing was conducted as a preliminary step to ensure the rigor of the analyses and the robustness of subsequent modeling results. First, all reverse-scored items in the questionnaire were identified and recoded to ensure directional consistency with forward-scored items.

Subsequently, scale-specific scoring procedures were applied to each measurement instrument—including the Physical Activity Rating Scale, Self-Control Scale, Emotion Regulation Questionnaire (ERQ), Zung Self-Rating Depression Scale (SDS), and Zung Self-Rating Anxiety Scale (SAS)—to ensure accurate computation and consistency of the derived variables.

#### 2.3.2 Feature engineering

Following data preprocessing, feature engineering was conducted based on the Multi-Process Action Control (M-PAC) model to ensure theoretically grounded feature construction. In line with the study’s focus on behavioral execution, features were derived primarily from the regulatory and reflexive processes, along with relevant contextual factors, and operationalized into computable variables for model development.

The extracted features included demographic and body-related variables (age, body weight), self-control dimensions (impulse control, healthy habits, resistance to temptation, work or study focus, and restraint in leisure activities), emotion regulation strategies (expressive suppression and cognitive reappraisal), as well as composite and subscale scores from the ERQ, SDS, and SAS.

To ensure data consistency and comparability across variables, items were scored following their original scale definitions, and all features were standardized to unified scoring directions prior to modeling. The mapping of theoretical constructs to operational definitions and corresponding computational features is presented in Table 1. The inclusion of psychological, behavioral, and body-related indicators was intended to reduce single-source measurement bias and enhance the robustness of the predictive model.

**Table 1:** Mapping between M-PAC theoretical processes and machine learning features.

M-PAC theoretical construct	Operational definition in this study	Corresponding ML features	Rationale for mapping	Implication for ML method choice
PAC process	Individuals’ capacity to regulate affective, motivational, and behavioral states during physical activity engagement	Impulse control ability Work focus ability Entertainment Restraint ability Emotional Suppression Level Cognitive Reappraisal Level	Behavioral execution support	Suggests non-linear and interaction-prone relationships, favoring models that can capture complex feature interactions
Reflective process	Cognitive evaluation of self-state, health consequences, and behavioral meaning	Health habits resistance to temptation Depression level SDS level	Cognitive evaluation	Supports inclusion of continuous psychological variables without assuming linear monotonic effects
Contextual factors	External and physiological conditions shaping behavior execution	Major Body weight Age	Boundary conditions	Justifies conditional decision structures, where contextual factors gate the influence of psychological variables
Behavioral outcome	Level of physical activity	Physical activity level	Observable behavioral outcome	Motivates classification-based models rather than regression

#### 2.3.3 Dataset partitioning, encoding, and addressing class imbalance

Categorical variables (e.g., major, SDS level, SAS level) were converted into numerical representations using label encoding. To prevent data leakage and ensure model generalizability, a strict preprocessing and validation pipeline was implemented.

##### 2.3.3.1 Initial train–test split

The dataset was randomly divided into a training set (80%) and an independent test set (20%) using a fixed random seed (random_state = 42). The test set was kept completely isolated and only used for final model evaluation.

##### 2.3.3.2 Class imbalance

Participants’ total physical activity scores (calculated from intensity, duration, and frequency) were categorized into low, medium, and high levels based on score distribution and scale-recommended thresholds, ensuring sufficient sample size in each category. Moderate imbalance was observed across classes. To address this, the SMOTE–Tomek hybrid method was applied to the training set only, generating synthetic samples for minority classes and removing Tomek links. The test set retained the original distribution.

##### 2.3.3.3 Feature scaling

Continuous features were standardized using parameters learned from the resampled training set. The same parameters were then applied to the test set, ensuring no information leakage. This pipeline guarantees unbiased model evaluation.

#### 2.3.4 Model selection and parameter optimization

To investigate the psychological factors underlying the intention–behavior gap in university students’ physical exercise, five commonly applied machine learning algorithms were selected for comparison: a multi-layer perceptron (MLP) classifier, XGBoost, random forest classifier, gradient-boosted decision trees (GBDT), and a decision tree classifier. The goal was to identify the algorithm with the best overall performance for constructing the final predictive model.

##### 2.3.4.1 Model selection rationale

The primary objective of this study was to model physical exercise behavior, which emerges from complex interactions among multidimensional psychological and physiological variables. Data derived from questionnaires grounded in the M-PAC model and stress process theory are inherently highly non-linear with complex interactions. Accordingly, tree-based ensemble models (random forest, XGBoost, and GBDT) were selected for their ability to capture non-linear effects and higher-order interactions in structured tabular data without strong parametric assumptions.

Beyond predictive accuracy, the study aimed to identify key influencing factors and assess their relative importance. Random forest and GBDT models provide stable and interpretable feature importance estimates, facilitating the identification of core predictors such as body weight and anxiety.

To examine marginal effects across different predictor ranges, particularly for high-intensity exercise participation, boosting-based models (especially GBDT) were employed due to their suitability for learning threshold-sensitive and piecewise relationships.

A decision tree model was included to provide a transparent hierarchical structure, allowing intuitive identification of dominant control variables (e.g., resistance to temptation) and their sequential relationships with exercise behavior, thereby facilitating interpretation within the M-PAC and stress process theory frameworks.

Finally, a multi-layer perceptron (MLP) model was incorporated to represent a contrasting paradigm based on distributed representations rather than explicit rule-based structures. This enabled assessment of whether predictive patterns from psychological and physiological variables were robust across fundamentally different algorithmic assumptions.

Collectively, these five algorithms address complementary analytical objectives—including predictive accuracy, interaction modeling, feature importance estimation, marginal effect exploration, and theoretical interpretability—reflecting a goal-oriented modeling strategy rather than reliance on a single algorithm.

##### 2.3.4.2 Parameter optimization

Hyperparameter tuning critically affects the predictive performance of machine learning models, enabling higher accuracy while mitigating overfitting. In this study, four widely used algorithms—Multi-Layer Perceptron (MLP), XGBoost, Random Forest, and Decision Tree—were optimized using GridSearchCV, which systematically explores predefined parameter spaces and evaluates all candidate combinations via cross-validation (Table 2).

**Table 2:** Optimized key parameter settings and results.

Model	Hyperparameter optimization space
Neural Network (MLP)	hidden_layer_sizes: [(50), (100), (50, 50)]
	alpha: [0.0001, 0.001]
	learning_rate_init: [0.001, 0.01]
XGBoost	n_estimators: [100, 200]
	max_depth: [3, 5, 7]
	learning_rate: [0.01, 0.1]
RandomForest	n_estimators: [100, 200]
	max_depth: [10, 15, None]
	min_samples_split: [2, 5]
	min_samples_leaf: [1, 2]
GBDT	n_estimators: [100, 200]
	learning_rate: [0.01, 0.1]
	max_depth: [3, 5]
DecisionTree	max_depth: [5, 10, 15, None]
	min_samples_split: [2, 5, 10]
	min_samples_leaf: [1, 2, 4]

The search spaces were defined based on three considerations: tuning only hyperparameters with substantial influence on model capacity and learning dynamics, referencing ranges commonly reported in prior studies and official documentation, and constraining ranges to balance computational feasibility with overfitting prevention given the moderate sample size. This strategy ensured stable, generalizable, and reproducible models while allowing sufficient exploration of the hyperparameter space.

#### 2.3.5 Model training

Following hyperparameter optimization, each model was trained independently as follows.

##### 2.3.5.1 Training data allocation

Eighty percent of the dataset was used for training, with the remaining 20 percent set aside for independent evaluation. To enhance reproducibility, a fixed random seed (42) was applied for dataset splitting and controlling stochastic elements across all algorithms.

##### 2.3.5.2 Model fitting

Each algorithm was trained on the training dataset, adjusting internal parameters to learn the relationships between features and outcomes. Training continued until reaching predefined stopping criteria, such as maximum iterations or convergence thresholds.

#### 2.3.6 Comprehensive model evaluation on the independent test set

After training the final models on the resampled training set with optimized parameters, performance was evaluated on the independent test set. Due to class imbalance, accuracy alone was deemed insufficient. Therefore, multiple evaluation metrics were reported.

##### 2.3.6.1 Class-specific metrics

Precision, recall (sensitivity), and F1-score were calculated for each physical activity level (low, moderate, high) to assess class-level performance.

##### 2.3.6.2 Aggregate metrics for model comparison

The weighted F1-score was adopted as the primary metric, balancing precision and recall while accounting for class imbalance. Macro-averaged AUC–ROC values were also computed using a one-vs-rest (OvR) strategy to evaluate.

##### 2.3.6.3 Overall metric

Accuracy was reported for reference but interpreted cautiously due to class imbalance. All results presented in the Results section were computed exclusively on the held-out test set, ensuring unbiased evaluation.

Statistical significance testing between models was not performed, as such tests are intended for inferential analysis rather than predictive modeling. Instead, performance was assessed using consistent data splits, cross-validated hyperparameter tuning, and multiple complementary metrics to ensure robust and fair comparison.

#### 2.3.7 Validation scope clarification

Generalizability in this study was evaluated through internal validation using an independent hold-out test set drawn from the same target population as the training data. All preprocessing, resampling, and hyperparameter optimization were performed on the training set after data partitioning. Consequently, the test set consists of unseen individuals from the same population, allowing an unbiased assessment of model reproducibility within this group. Thus, reported performance reflects the model’s ability to generalize to new individuals with similar characteristics, rather than to entirely different populations.

## 3 Evaluation findings and analysis

### 3.1 Comparative analysis of classification performance in machine learning algorithms

**Figure 1:**
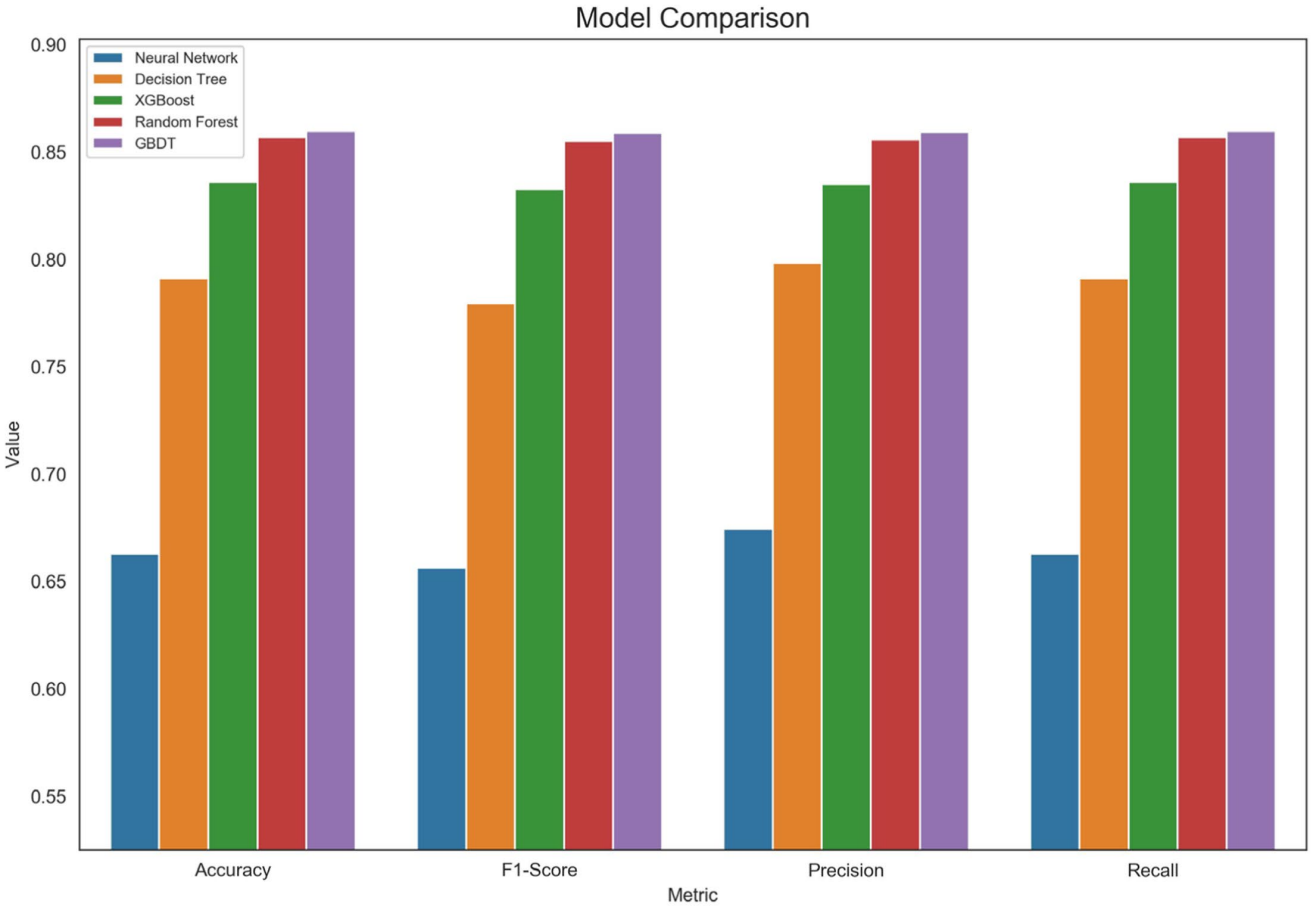
Summary chart of evaluation indicator results.

The evaluation results (Figure 1) demonstrate that the GBDT model performs exceptionally well across all metrics, achieving the highest values particularly in accuracy (0.8597) and F1 score (0.8589). This indicates that the GBDT model not only accurately classifies samples overall but also excels at balancing precision and recall. XGBoost and Random Forest models followed in performance, indicating their efficacy in handling classification tasks, though slightly inferior to GBDT in overall capability. In contrast, neural networks and decision tree models demonstrated comparatively weaker performance, potentially exhibiting limitations when classifying complex data. Overall, the GBDT model’s superior performance underscores its enhanced robustness and generalization capability when processing non-linear relationships and complex data features.

### 3.2 Receiver operating characteristic curve and confusion matrix analysis

**Figure 2:**
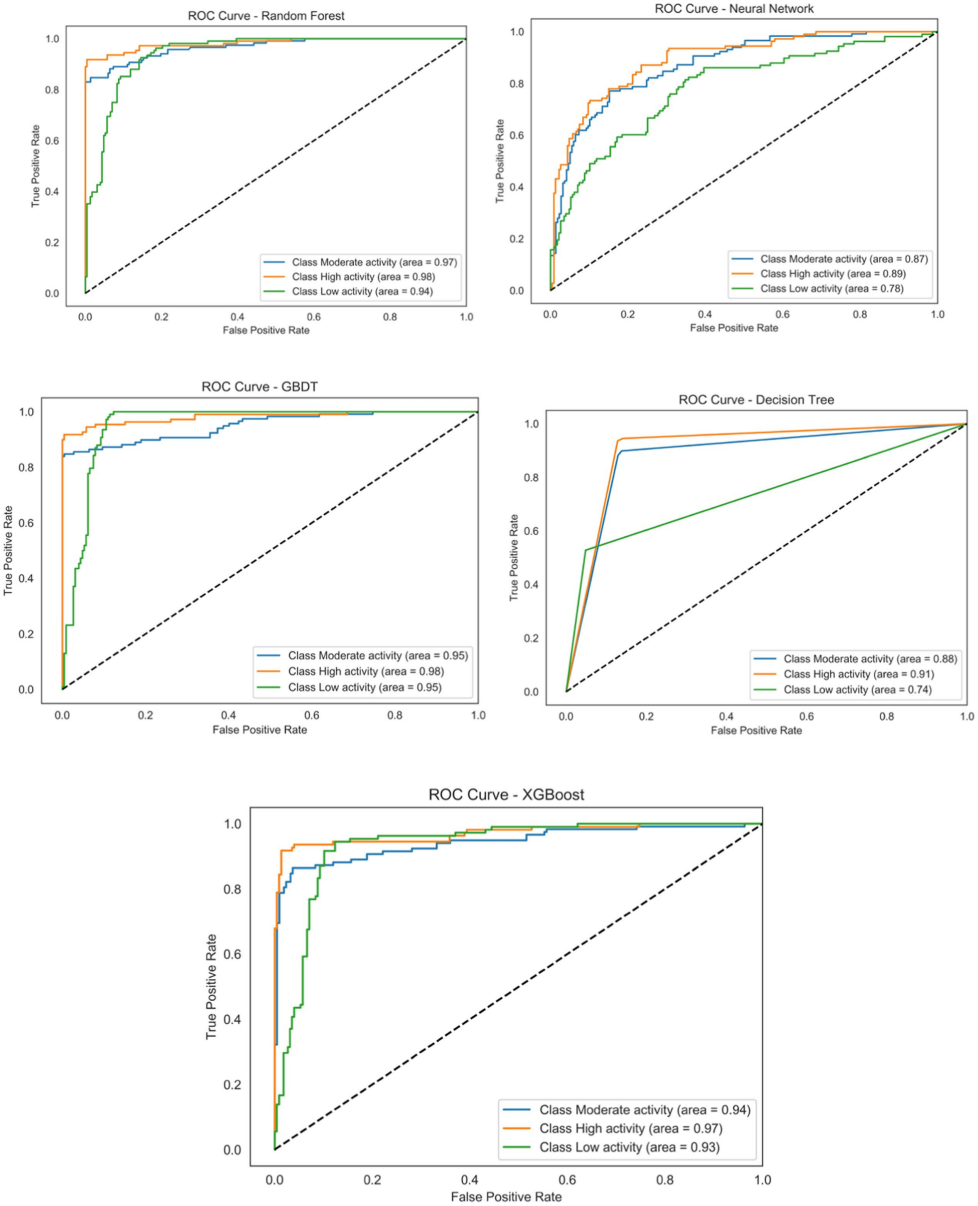
ROC curve diagram.

When evaluating the classification performance of various models, ROC curves and AUC (area under the curve) values provide crucial reference points, particularly in assessing a model’s ability to distinguish positive and negative classes under different thresholds (Figure 2). Among these, the GBDT model demonstrated the most favorable ROC curve performance, achieving an AUC value of 0.95. Detailed analysis reveals that this model demonstrates exceptional discriminatory power across both the ‘high physical activity’ and ‘low physical activity’ categories, achieving a favorable balance between TPR (true positive rate) and FPR (false positive rate). Within the ‘high physical activity’ category, the GBDT model’s TPR approaches 0.94, while its FPR remains below 0.05, indicating remarkably high accuracy when handling these classifications.

It is important to note that these findings are based on internal validation within the sampled population. Therefore, interpretations regarding the model’s robustness or generalizability should be made with appropriate caution. Further research incorporating external validation or more stringent validation strategies would help to establish the broader applicability of the model.

### 3.3 Confusion matrix analysis

**Figure 3:**
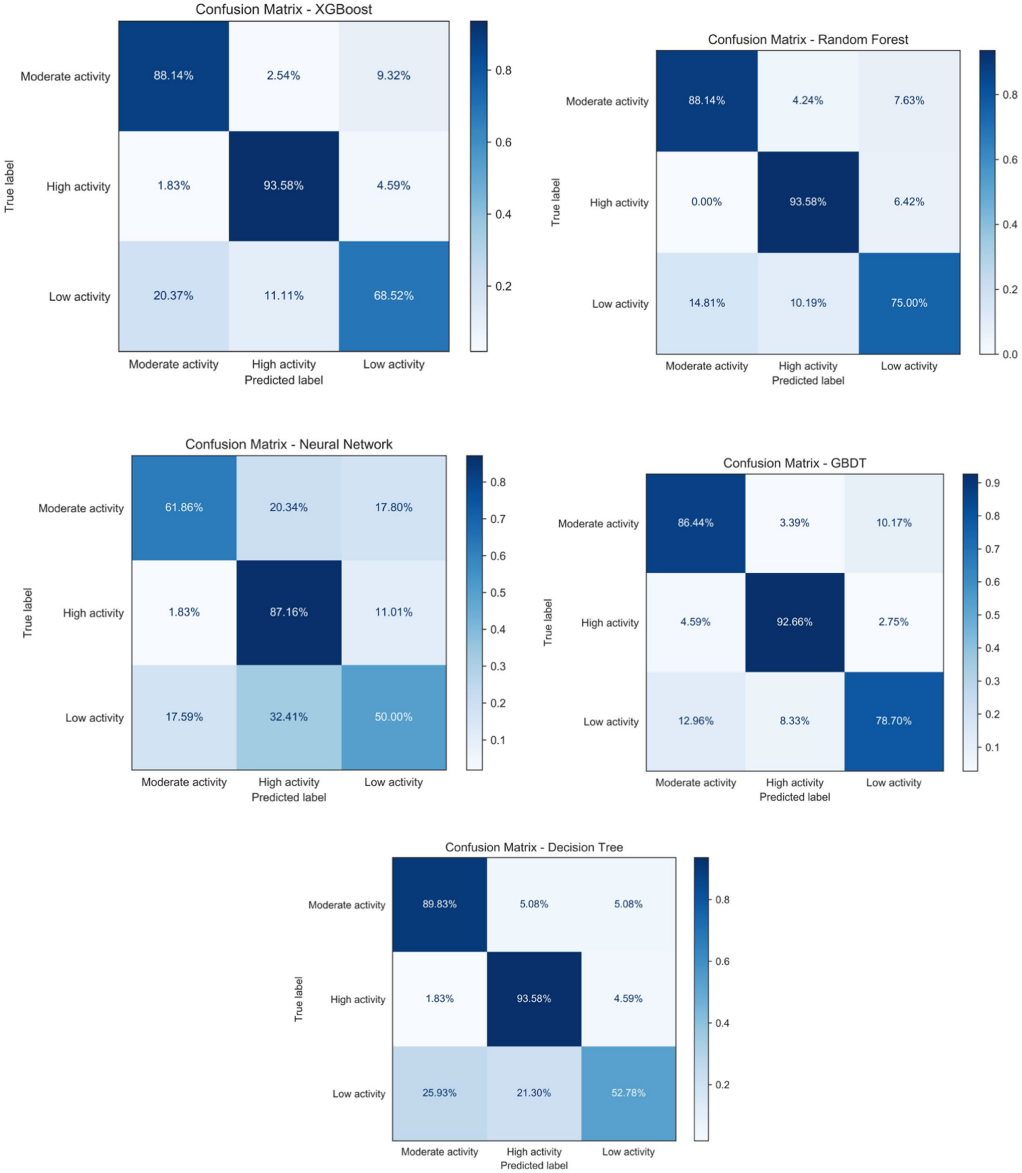
Confusion matrix analysis.

The confusion matrices illustrate model performance across physical activity categories and reveal consistent misclassification patterns (Figure 3). Classification was most accurate for the low- and high-activity groups, reflecting distinct feature profiles, while misclassifications occurred predominantly in the moderate activity group. For the GBDT model, although the high-activity category achieved a 94% correct classification rate, most misclassified samples were assigned to the moderate category. Similar trends were observed for XGBoost and Random Forest, where low-activity samples were more often misclassified as moderate rather than high.

These patterns reflect the intermediate and heterogeneous nature of moderate activity, whose psychological, behavioral, and self-regulatory characteristics overlap with both low- and high-activity groups, resulting in less distinct decision boundaries. The neural network model exhibited a higher tendency to misclassify moderate activity as high, while the decision tree showed elevated misclassification rates for high activity, suggesting that single-tree structures struggle to capture complex interactions near category boundaries. Notably, direct misclassification between low and high activity was rare, indicating that extreme behavioral states were effectively distinguished.

Overall, these results highlight the continuous nature of physical activity and the challenges of discretizing it into ordinal categories. In combination with ROC and AUC analyses, the GBDT model demonstrated superior classification performance and generalizability, making it the optimal model for this study.

### 3.4 Feature importance analysis

**Figure 4:**
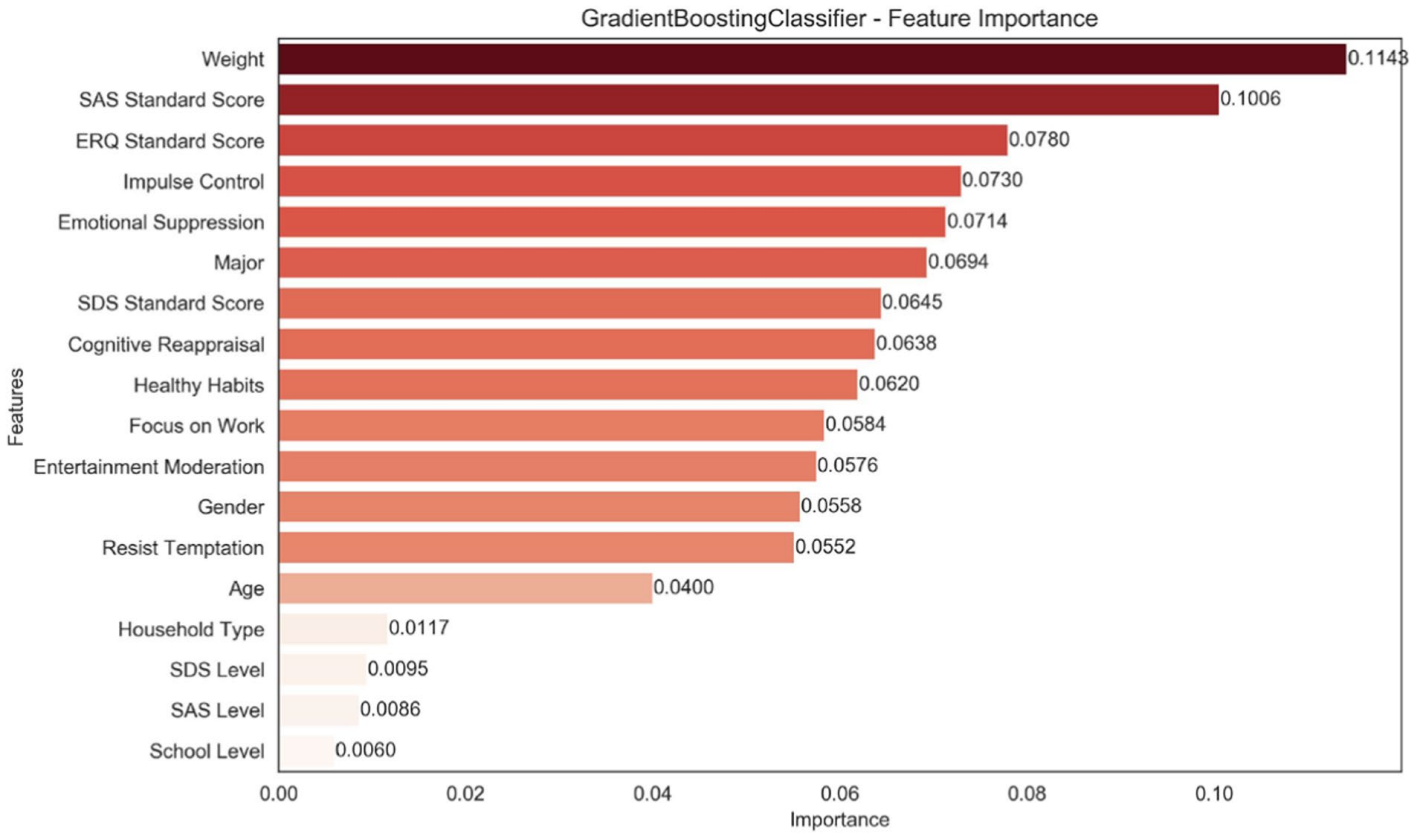
Feature importance analysis chart.

Feature importance analysis was conducted to examine the relative contributions of all predictors in the GBDT model (Figure 4). Discussion focuses on the most influential features and interprets them within established behavioral frameworks.

Body weight emerged as the most important predictor (importance = 0.1143). Measured in kilograms as a raw physiological indicator, body weight reflects embodied constraints such as physical load, movement efficiency, perceived exertion, and exercise-related self-efficacy, which can shape affective experiences and perceived difficulty during activity. Within the M-PAC framework, body weight acts as a distal or contextual factor influencing behavioral execution through perceived effort and emotional responses, rather than as a direct motivational driver.

Among psychological variables, the SAS standard score ranked second (importance = 0.1006), highlighting the role of anxiety in exercise participation. Elevated anxiety may increase avoidance, cognitive load, and perceived barriers, thereby interfering with the intention–behavior transition, consistent with stress process theory and the M-PAC framework. The ERQ standard score, representing emotion regulation capacity, ranked third (importance = 0.0780), reflecting the self-regulatory pathway of M-PAC. Effective emotion regulation enables individuals to manage discomfort, negative affect, and stress, supporting persistence and adherence to exercise.

It is important to note that feature importance in tree-based models reflects relative contributions to predictive accuracy, not causal effects. Although no single psychological variable exceeded body weight individually, their combined influence accounted for a substantial portion of the model’s explanatory capacity. This aligns with theoretical models emphasizing that exercise behavior emerges from the joint effects of physiological constraints, affective responses, and self-regulatory capacities. Overall, the ranking supports a multi-component interpretation in which physiological conditions provide the contextual baseline, while psychological and self-regulatory processes govern behavioral execution and maintenance.

### 3.5 Dependency graph analysis

Partial dependence plots (PDPs) were generated for each feature to examine their marginal effects on the model’s predicted physical activity levels. This analysis illustrates how variations in individual features influence predicted outcomes across their numerical ranges, highlighting non-linear relationships and potential threshold effects within the GBDT model. By visualizing these trends, the PDPs provide insight into how physiological, psychological, and self-regulatory factors contribute to exercise behavior in a quantifiable and interpretable manner.

#### 3.5.1 Partial dependence plots for demographic variables

**Figure 5:**
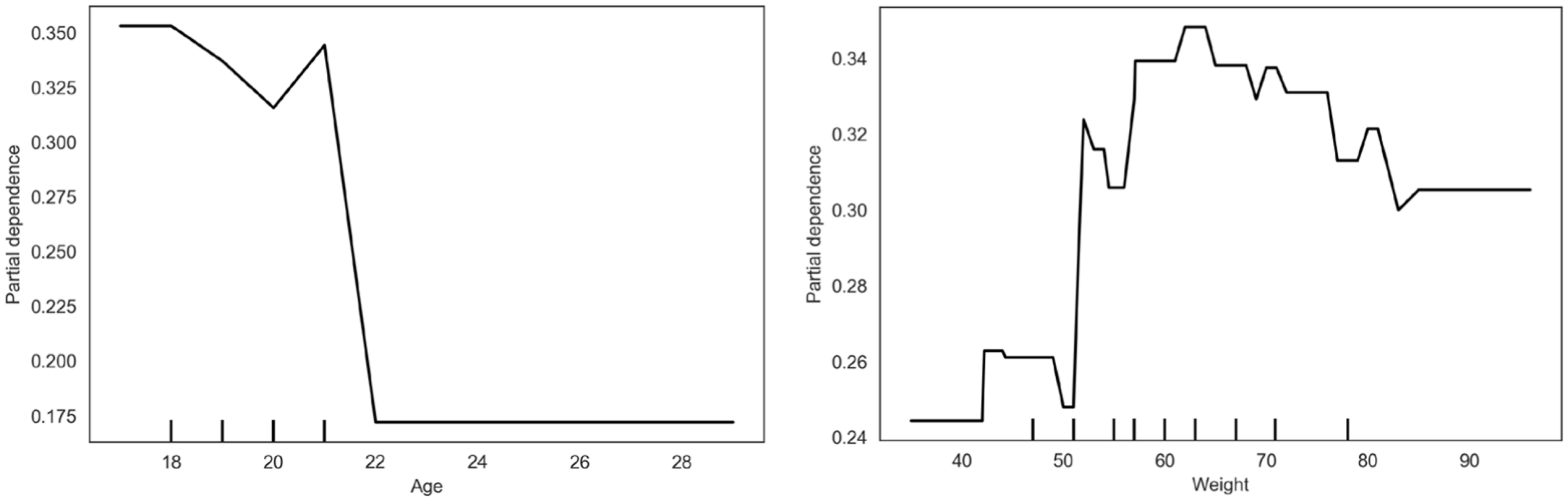
Dependence on demographic bias.

Partial dependence plots for age and weight (Figure 5) reveal distinct patterns in predicted high-intensity exercise. The age plot shows a gradual decline in predicted probability from 0.35 at age 18 to 0.175 at age 22, indicating younger individuals are more likely to engage in high-volume exercise. After age 22, the probability decreases more sharply, suggesting that reduced physical capacity or lifestyle changes may limit high-intensity exercise among older cohorts.

The weight plot exhibits a non-linear relationship with high-intensity exercise. For individuals below 50 kg, the predicted probability remains low (0.24). Between 50 and 80 kg, the probability increases to ~0.34 and stabilizes, indicating a higher likelihood of engaging in high-volume exercise within this weight range. Beyond 80 kg, the predicted probability slightly declines. This pattern reflects the model’s predicted tendencies across different weight ranges without implying causal relationships.

#### 3.5.2 Self-control dependency diagram

**Figure 6:**
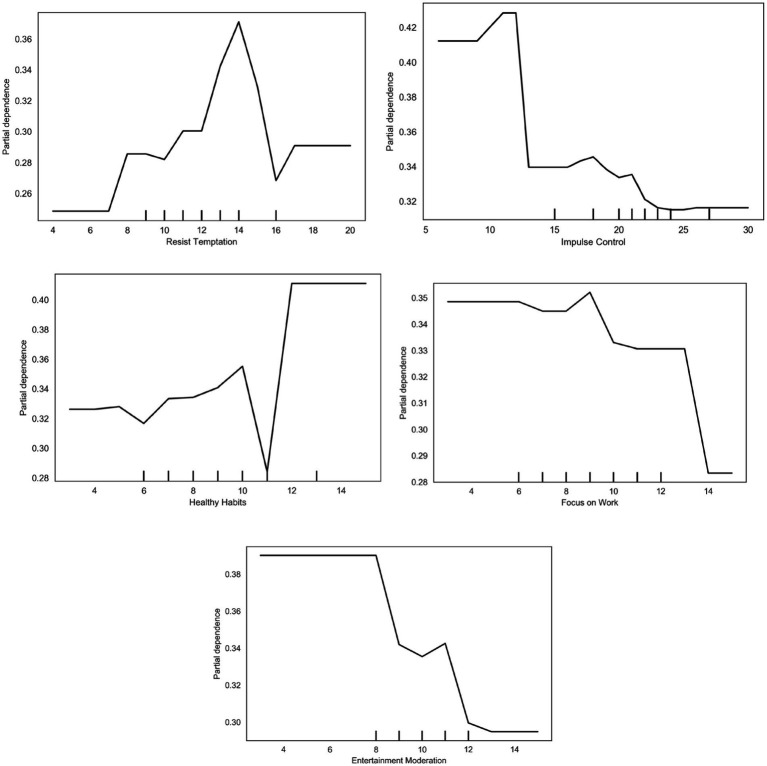
Self-control dependency diagram.

The partial dependence plot for impulse control (Figure 6) shows a non-linear pattern in the predicted probability of high-intensity exercise. At lower scores (below ~10), predicted probabilities are relatively high, then decline through the mid-range and stabilize at higher levels, decreasing from approximately 0.42 to 0.32. This pattern reflects the model’s learned tendencies rather than causal effects. In this study, impulse control primarily captures inhibitory regulation—the ability to suppress immediate urges—rather than proactive motivation, habitual behaviors, or identity-based regulation, highlighting the multidimensional nature of self-control.

Within the M-PAC framework, impulse control primarily reflects inhibitory regulation rather than a direct motivational driver. High-intensity exercise involves multiple factors, including affective responses, habitual routines, and identity-consistent regulation. The partial dependence pattern suggests that impulse control interacts with these factors, functioning more as a context-dependent constraint than as a standalone predictor of exercise behavior. This relationship is non-linear and reflects the model’s learned tendencies rather than implying that higher impulse control reduces exercise participation.

Other self-control–related dimensions exhibited distinct and non-linear patterns. Healthy habits were positively associated with predicted high-intensity exercise, with partial dependence values increasing at higher score levels, suggesting a facilitating role for habitual regulation. Resistance to temptation displayed a non-monotonic pattern, with predicted probabilities rising up to mid-range scores before slightly declining, indicating interactions with other behavioral factors. In contrast, focus on work and leisure moderation showed predominantly negative associations with high-intensity exercise. From a time- and resource-allocation perspective, elevated work focus reflects sustained cognitive engagement and prioritization of academic or task-related demands, which may coincide with prolonged sedentary time or cognitive fatigue, thereby limiting the model-predicted probability of vigorous activity.

Within the M-PAC framework, such contextual factors are considered distal conditions that influence behavioral enactment rather than direct motivational drivers. The observed negative associations reflect context-dependent, non-linear relationships learned by the predictive model rather than causal effects. These patterns highlight how competing demands and cognitive load modulate the expression of exercise-related behaviors, consistent with prior research indicating that elevated role demands are associated with lower engagement in physical activity despite positive health intentions.

Overall, these heterogeneous patterns highlight the value of treating self-control as a multidimensional construct. The partial dependence results indicate that different self-regulatory components are associated with exercise behavior through distinct patterns, consistent with theoretical models emphasizing the combined influence of physiological conditions, affective processes, habits, and self-regulatory capacities (see Figure 6).

#### 3.5.3 Emotional regulation dependency diagram

**Figure 7:**
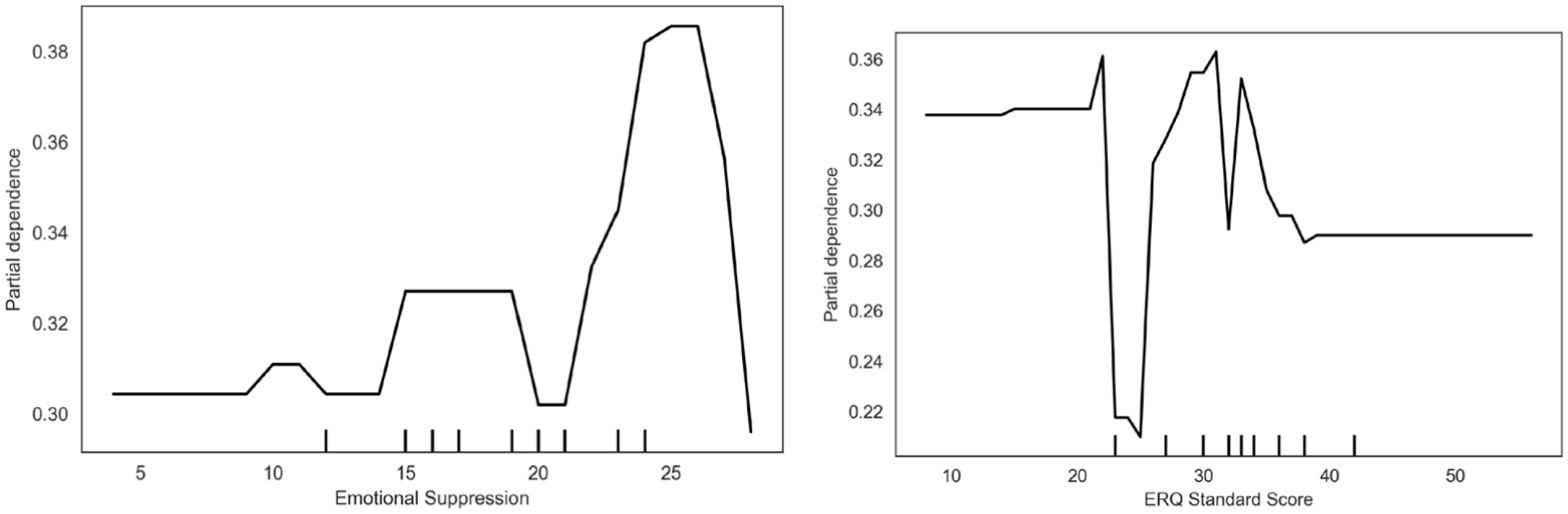
Emotional regulation dependency.

The partial dependence plot for Emotional Suppression (Figure 7) reveals that scores above 20 exhibit a significant increase in partial dependence values to approximately 0.38, indicating that individuals with high emotional suppression are more inclined toward engaging in vigorous exercise. However, at lower scores (<10), the bias dependency value remains around 0.30, indicating a reduced tendency toward high-intensity exercise. This may relate to the complex effects of emotional suppression strategies on mood and behavior. The bias dependency plot for Cognitive Reappraisal shows that higher-scoring individuals (20) are more inclined toward high-intensity exercise, with bias dependency values exceeding 0.40. Conversely, individuals with lower scores (<10) exhibited bias values fluctuating between 0.25 and 0.32, indicating a reduced propensity for high-intensity exercise. This suggests that stronger cognitive reappraisal abilities correlate with higher levels of exercise participation.

#### 3.5.4 Depression and anxiety dependency diagram

**Figure 8:**
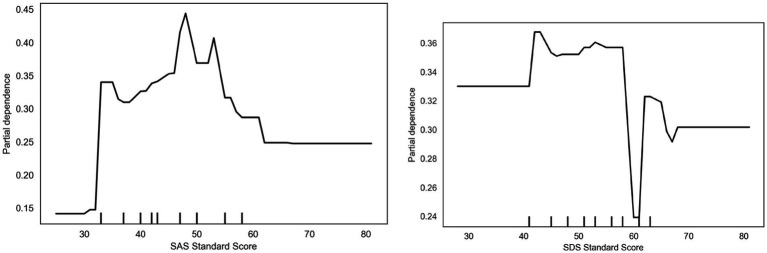
Depression and anxiety dependency.

The skewness dependence plot for the ERQ Standard Score (Figure 8) indicates that when scores fall between 20 and 30, the skewness value markedly decreases to approximately 0.22, reflecting a low propensity for participation in vigorous physical activity. However, when scores exceed 30, the skewness value gradually recovers to above 0.30, suggesting that higher ERQ scores correlate with a greater tendency toward physical activity participation. The bias dependence plot for the SDS Standard Score indicates that individuals with higher depression scores (60) exhibit reduced participation in vigorous exercise, with bias dependence values falling below 0.30. Conversely, between scores of 40–50, the partial dependence value increased to 0.36, indicating a higher propensity for physical activity participation. This may reflect behavioral patterns where some mildly depressed individuals regulate their mood through exercise. The bias dependence plot for the SAS Standard Score (SAS) indicates that individuals with high anxiety scores (60) exhibit a significantly reduced tendency toward high-intensity exercise, with the bias dependence value falling below 0.25. Conversely, scores between 40 and 50 yielded a partial dependence value of approximately 0.40, suggesting individuals with moderate anxiety levels may be more inclined to release stress through high-intensity exercise. This phenomenon indicates a complex relationship between anxiety levels and exercise behavior.

In summary, this study applied multiple machine learning models to behavioral prediction, aiming to construct an optimal predictive model through hyperparameter tuning, feature importance analysis, and comprehensive performance evaluation. Data quality and model training were first ensured through thorough preprocessing and feature engineering. Subsequently, five models—neural networks, XGBoost, random forests, gradient-boosted decision trees (GBDT), and decision trees—were systematically trained and evaluated.

Model evaluation indicated that the GBDT model achieved the highest performance across key metrics, including accuracy and F1 score, demonstrating strong robustness and generalizability, and was therefore identified as the optimal classifier. XGBoost and Random Forest followed closely, showing strong but slightly lower performance in handling complex classification tasks. In contrast, neural networks and decision trees exhibited comparatively weaker performance, reflecting limitations in capturing non-linear relationships and complex feature interactions.

Feature importance analysis highlighted weight, SAS standard score, and ERQ standard score as the most influential predictors, underscoring their substantial contribution to the model’s predictive capacity. Partial dependence analyses further illustrated how individual features shape model predictions across different value ranges. In particular, age, weight, impulse control, and emotion regulation exhibited distinct patterns in predicting tendencies toward high-volume exercise.

Overall, this study not only established an effective behavioral prediction model but also provided interpretable insights into its decision-making processes through detailed feature-level analyses.

### 3.6 Classification decision tree model

**Figure 9:**
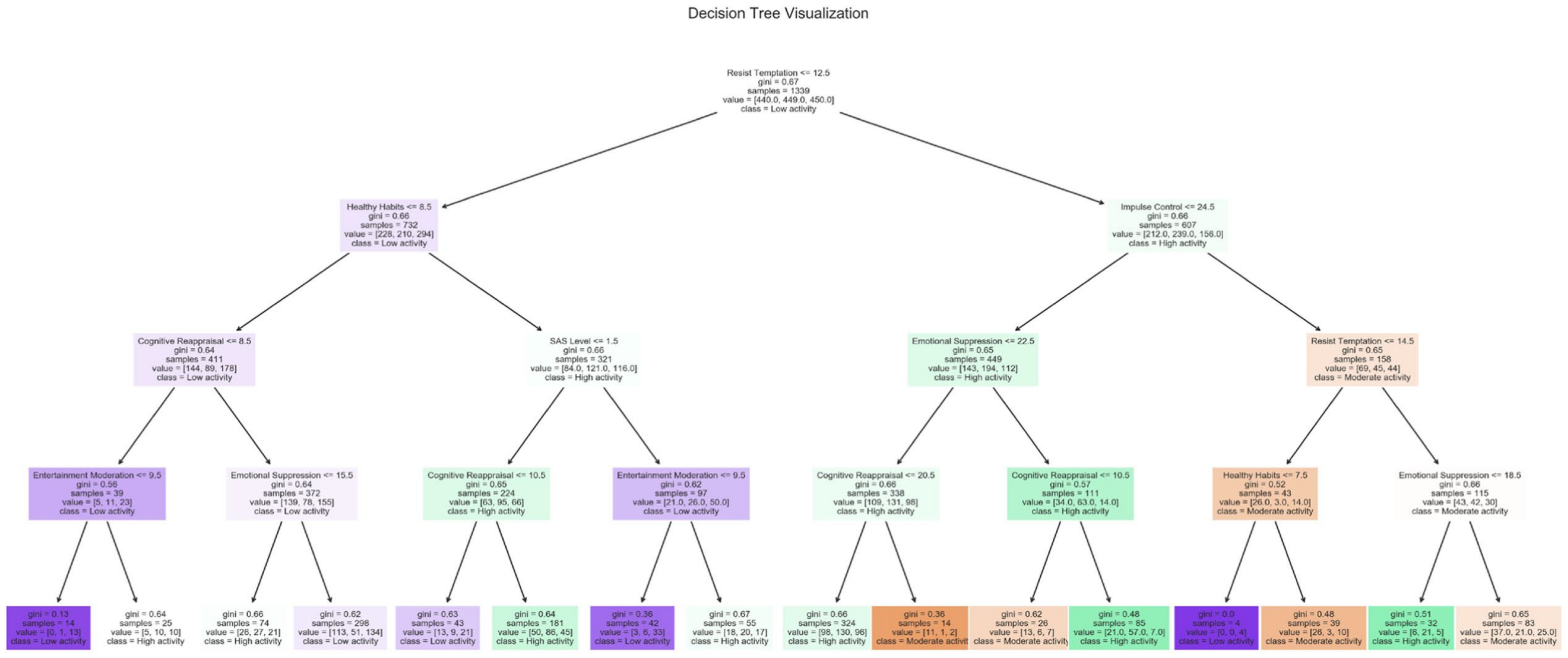
Classification decision tree model.

The distribution of leaf node categories indicates that distinct decision pathways yield predictions for three levels of physical activity. High activity outcomes (green nodes) predominantly emerge from specific pathways associated with stronger self-regulatory capacity and more adaptive emotion regulation strategies. In contrast, prediction pathways for low activity levels (purple nodes) are relatively dispersed, suggesting that multiple combinations of factors may contribute to insufficient physical activity participation. Moderate activity levels (orange nodes) exhibit the most widespread distribution, reflecting the heterogeneity of individual characteristic configurations encompassed within this category. The decision tree model was employed as an interpretable, rule-based approach to illustrate hierarchical interaction patterns among key predictors of physical activity. Importantly, the tree is not intended to establish causal relationships, but rather to complement the partial dependence plot (PDP) analysis by revealing conditional decision pathways learned by the classification model. To enhance interpretability and reduce overfitting, the original tree was pruned using cost-complexity pruning, retaining only splits that contributed meaningfully to classification performance. The final tree consists of a reduced number of layers with clearly defined decision paths. Sample sizes for all internal and leaf nodes are reported in Figure 9 to improve transparency and facilitate evaluation of node stability.

The Gini index was adopted as the splitting criterion, given its widespread use in multi-class classification trees, computational efficiency, and consistency with other tree-based models applied in this study (e.g., gradient boosting). Lower Gini values indicate higher node purity, enabling effective separation of activity-level categories. In the resulting tree, resistance to temptation emerges as the root node, highlighting its central role in differentiating physical activity levels across the sample. This finding is consistent with self-control theory, which emphasizes resistance to immediate temptation as a foundational component of health-promoting behavior. Subsequent splits reveal two broad conditional pathways. Within the lower resistance-to-temptation subgroup, health habits play a prominent role in further classification, suggesting that habitual regulation becomes particularly salient when inhibitory self-control is weaker. Within this pathway, anxiety levels and cognitive reappraisal ability provide additional differentiation, indicating that affective states and emotion regulation strategies modulate exercise behavior under constrained self-regulatory conditions. In contrast, among individuals with higher resistance to temptation, impulse control and emotional suppression emerge as more salient classifiers. This pattern suggests that when baseline self-regulatory capacity is relatively strong, finer distinctions in inhibitory control and emotion regulation contribute to variability in exercise participation. Cognitive reappraisal appears across multiple branches of the tree, reflecting its context-dependent role in shaping behavioral outcomes rather than a uniform marginal effect.

The distribution of leaf nodes further indicates that high physical activity levels are primarily associated with specific configurations characterized by strong self-regulatory capacity combined with adaptive regulation strategies. In contrast, low and moderate activity levels arise from a broader range of factor combinations, underscoring the heterogeneity of behavioral pathways leading to insufficient physical activity. When considered alongside the PDP results, the decision tree does not contradict the observed marginal associations. Instead, it illustrates how variables that exhibit weak or even negative average effects in PDP analyses (e.g., impulse control or work focus) may still function as critical split variables under specific conditional contexts. Together, these complementary analyses highlight the multidimensional and interaction-dependent nature of self-regulation in physical activity behavior, aligning with theoretical perspectives that emphasize the joint influence of habits, affective processes, and self-regulatory mechanisms.

## 4 Discussion

### 4.1 The impact of machine learning on predicting participation in sporting activities

The application of machine learning techniques in education and health promotion has advanced rapidly, providing novel opportunities to identify behavioral correlates through efficient data modeling. In this study, multiple machine learning algorithms were employed to construct predictive models of university students’ participation in physical activity, incorporating demographic characteristics, self-control sub-dimensions, emotion regulation strategies, and psychological indicators such as depression and anxiety. Across the evaluated models, the Gradient Boosted Decision Tree (GBDT) demonstrated superior performance on key evaluation metrics. In particular, it achieved the highest F1 score (0.8589), indicating strong overall predictive performance. This advantage likely reflects several methodological properties of the algorithm. First, GBDT is well suited to capturing complex nonlinear relationships and interaction patterns among variables, which is particularly advantageous in multidimensional datasets with limited sample sizes and moderate class imbalance. Second, the model exhibits strong adaptability to high-dimensional feature spaces, enabling the identification of informative feature combinations without requiring explicit specification. In addition, the ensemble structure of tree-based learners offers a practical balance between predictive accuracy and interpretability, facilitating the examination of psychologically relevant variables such as resistance to temptation and cognitive reappraisal within the predictive framework. Results from the ROC curve analysis further suggest that the model’s classification performance reflects the discriminative capacity of the selected feature set, encompassing both demographic and psychological dimensions. Collectively, these findings indicate that self-control capacities, emotion regulation strategies, and affective states (e.g., depression and anxiety) are meaningfully associated with variations in physical activity participation among university students. At the same time, it should be acknowledged that other influential factors not included in the present models—such as social environment, campus sports culture, curriculum structure, and peer influences—may also be relevant to physical activity behavior. Accordingly, while the GBDT model demonstrated high predictive accuracy within the current dataset, future research may benefit from expanding the range of explanatory variables, refining feature engineering strategies, and incorporating longitudinal or time-dynamic data to improve generalizability and practical applicability. Overall, the findings support the utility of machine learning approaches—particularly GBDT—in modeling physical activity participation among university students, while offering methodological insight for the development of more targeted health promotion strategies. It should be emphasized that the present study adopts a cross-sectional, observational design. As such, all relationships identified by the machine learning models represent predictive associations within the sampled data rather than causal effects. References to predictive importance or classification performance are intended to describe model behavior and should not be interpreted as evidence of temporal precedence or causal influence. The results therefore provide a descriptive, hypothesis-generating mapping of factors associated with physical activity behavior, rather than causal validation of underlying theoretical mechanisms.

### 4.2 Effective intervention pathways for physical exercise behavior

The physical fitness of university students is closely linked to healthy lifestyle habits, and scientific assessments of physical condition are commonly used to characterize exercise participation patterns. The development of regular physical exercise habits is shaped by both individual characteristics and environmental conditions, including the availability of supportive sporting environments. However, contemporary university students face substantially more competing demands than previous generations, particularly those associated with short-video consumption, online gaming, and fragmented online social interactions. These factors are frequently accompanied by reduced time allocation and attentional resources for physical activity, coinciding with generally insufficient levels of exercise participation (Yipeng, 2015). The findings of this study indicate that physical exercise behavior is positively correlated with multiple sub-dimensions of self-control. These associations suggest that self-control resources are meaningfully related to exercise behavior, particularly in situations characterized by competing demands and immediate temptations (Rouse et al., 2013). Individuals reporting higher levels of self-control also tend to report greater consistency in exercise participation, which may reflect differences in behavioral regulation strategies rather than uniform effects across all self-control components.

With respect to emotion regulation, prior research has consistently identified cognitive reappraisal as an emotion regulation strategy that is closely associated with more favorable exercise-related experiences. Evidence suggests that the use of cognitive reappraisal during physical activity is associated with lower perceived exertion and reduced emotional arousal, alongside enhanced exercise enjoyment (Giles et al., 2018). In addition, cognitive reappraisal has been linked to more positive exercise-related attitudes, stronger intrinsic motivation, and higher behavioral intention in college student populations (He et al., 2022). Within the context of the present study, these findings support the interpretation of emotion regulation strategies—particularly cognitive reappraisal—as psychologically relevant correlates of exercise behavior, rather than as empirically validated intervention components.

It should also be noted that depression, anxiety, emotion regulation difficulties, and reduced self-control resources frequently co-occur and are jointly associated with physical activity behavior in existing research. Rather than representing independent or unidirectional causal mechanisms, these psychological characteristics may form overlapping profiles that correspond to different patterns of exercise participation. From this perspective, stronger emotion regulation capacity and self-control resources may be understood as markers associated with more stable exercise behavior, without implying a direct causal pathway.

In summary, informed by the Multi-Process Action Control (M-PAC) model and stress process theory, the present study highlights the value of incorporating psychological variables into predictive analyses of physical exercise behavior. The findings provide descriptive and hypothesis-generating reference points for future longitudinal and intervention-based research aimed at clarifying the temporal ordering, contextual conditions, and functional roles of self-control and emotion regulation within the intention–behavior–habit process.

### 4.3 Enrichment and extension of existing theoretical models of physical exercise behavior

In exercise behavior research, the Theory of Planned Behavior (TPB) (Ajzen, 2011) and the Health Belief Model (HBM) (Xinying and Guoyan, 2009) are widely used frameworks for understanding health-related decision-making. By emphasizing proximal cognitive determinants (e.g., attitudes and perceived control) and more distal belief-based factors (e.g., perceived barriers and severity), these models provide a conceptual foundation for examining the intention–behavior relationship in physical activity.

Building on this tradition, the present study situates exercise participation within the intention–behavior conversion pathway and is consistent with recent evidence showing that perceived barriers, perceived severity, and mobile media usage are statistically associated with variation in the intention–behavior gap (Jingyi and Sheng, 2024). The findings further highlight the relevance of attitudinal and regulatory variables, such as emotion regulation strategies, in relation to exercise behavior, supporting the continued applicability of TPB-related constructs without implying causal effects.

By integrating the Multi-Process Action Control (M-PAC) model with Stress Process Theory, this study adopts a multi-level perspective to examine the joint associations of demographic, physical, and psychological variables with physical exercise behavior. The findings indicate that, despite widespread exercise intentions, sustained participation reflects a complex and non-linear process shaped by multiple co-occurring constraints rather than any single factor.

These results align with action-oriented frameworks, such as Action Stage Theory, which emphasize the intention–action transition (the Rubicon process), and suggest that different configurations of psychological and contextual factors may correspond to distinct behavioral pathways. Theoretically, the study extends intention-based models by highlighting the context- and stage-dependent relevance of emotion regulation and self-control, supporting the value of multi-stage and branching decision-path approaches in future research.

One notable finding is that body weight emerged as an influential predictor across multiple models, despite the study’s primary focus on psychological variables. This result warrants cautious interpretation. From a methodological perspective, feature importance estimates in tree-based algorithms are partly shaped by the structural properties of predictors. In particular, impurity-based importance measures may assign greater importance to continuous variables due to their increased flexibility for split optimization, rather than solely reflecting substantive explanatory dominance.

From a theoretical standpoint, body weight may be more appropriately understood as a distal contextual condition within the M-PAC framework, influencing behavioral regulation demands rather than directly determining behavior. Furthermore, given the cross-sectional design, the observed association is better interpreted as reflecting the dynamic interplay between physical and psychological processes, rather than implying a causal effect.

## 5 Research limitations

The present study has several limitations that should be acknowledged.

First, the use of convenience sampling limits external validity. Although participants were recruited from multiple provinces to reduce local bias, the absence of probabilistic sampling restricts generalizability. Given the sensitivity of machine learning models to sample-specific distributions, the identified predictive structures may partly reflect contextual characteristics of the sampled institutions rather than stable population-level patterns. Replication using stratified sampling and independent datasets is therefore necessary.

Second, the findings should be interpreted within their cultural and institutional context. All participants were university students from mainland China, where physical activity behaviors are shaped by specific sociocultural norms and educational environments. Psychological correlates such as self-control and emotion regulation may manifest differently across cultures, limiting direct cross-national generalization.

Third, all variables were assessed using self-report questionnaires. Despite the use of validated instruments, self-report data are subject to recall and social desirability biases, particularly for physical activity behaviors that are socially valued. Such biases may introduce measurement error and contribute to misclassification in activity levels.

Fourth, physical activity was measured using a questionnaire-based categorical approach rather than objective or fine-grained monitoring. While common in large-scale studies, this method may not fully capture variability in intensity, timing, or context. Future research would benefit from incorporating wearable or ecological momentary assessment methods.

Fifth, the cross-sectional design precludes any inference regarding temporal ordering or causality. The identified relationships should therefore be interpreted strictly as predictive associations, with longitudinal or experimental designs required to clarify directionality.

Sixth, although strict train–test splitting was applied, no independent external validation sample was included. As a result, the robustness of the models beyond the current dataset remains uncertain and should be tested across independent cohorts.

Finally, the use of SMOTE to address class imbalance may have introduced artificial patterns into the training data. Although applied only to the training set, some learned decision boundaries may partially reflect algorithm-induced structures rather than naturally occurring behavioral patterns. Caution is therefore warranted, and future studies should compare alternative imbalance-handling strategies or rely on larger, more balanced datasets.

## 6 Conclusion

Grounded in the Multi-Process Action Control (M-PAC) model and stress process theory, this study integrates demographic and psychological variables to construct predictive models of physical exercise behavior among university students, with an emphasis on identifying associative patterns between intention-related psychological factors and observed exercise participation.

Among the five machine learning algorithms compared, the Gradient Boosted Decision Tree (GBDT) demonstrated the strongest classification performance. Model interpretability analyses indicated that body weight, age, depression and anxiety symptoms, self-control sub-dimensions, and emotion regulation strategies were strongly associated with variations in exercise behavior, illustrating how different psychological and demographic features contribute to model predictions across value ranges without implying causal relationships.

In addition, classification tree results revealed conditional association structures among psychological variables, with certain self-control components emerging as salient splitting features. These structures reflect the internal logic of the predictive models and should be interpreted as descriptive associations rather than behavioral pathways.

Overall, the findings highlight the value of machine learning approaches for mapping complex and non-linear associations within the exercise intention–behavior context and provide hypothesis-generating reference points for future longitudinal and intervention-based research.

## Data Availability

The original contributions presented in the study are included in the article/supplementary material, further inquiries can be directed to the corresponding author.
